# ProTAME Arrest in Mammalian Oocytes and Embryos Does Not Require Spindle Assembly Checkpoint Activity

**DOI:** 10.3390/ijms20184537

**Published:** 2019-09-13

**Authors:** Lenka Radonova, Tereza Svobodova, Michal Skultety, Ondrej Mrkva, Lenka Libichova, Paula Stein, Martin Anger

**Affiliations:** 1 Central European Institute of Technology, Department of Genetics and Reproduction, Veterinary Research Institute, 621 00 Brno, Czech Republic; radonova@vri.cz (L.R.); svobodova@vri.cz (T.S.); skultety@vri.cz (M.S.); mrkva@vri.cz (O.M.); lenlibichova@gmail.com (L.L.); 2 Cellular Imaging Core Facility, Central European Institute CEITEC Masaryk University, 624 00 Brno, Czech Republic; 3 National Institute of Environmental Health Sciences, NIH, Durham, NC 27709, USA; paula.stein@nih.gov

**Keywords:** cell cycle, oocytes, meiosis, proTAME, anaphase promoting complex, spindle assembly checkpoint

## Abstract

In both mitosis and meiosis, metaphase to anaphase transition requires the activity of a ubiquitin ligase known as anaphase promoting complex/cyclosome (APC/C). The activation of APC/C in metaphase is under the control of the checkpoint mechanism, called the spindle assembly checkpoint (SAC), which monitors the correct attachment of all kinetochores to the spindle. It has been shown previously in somatic cells that exposure to a small molecule inhibitor, prodrug tosyl-l-arginine methyl ester (proTAME), resulted in cell cycle arrest in metaphase, with low APC/C activity. Interestingly, some reports have also suggested that the activity of SAC is required for this arrest. We focused on the characterization of proTAME inhibition of cell cycle progression in mammalian oocytes and embryos. Our results show that mammalian oocytes and early cleavage embryos show dose-dependent metaphase arrest after exposure to proTAME. However, in comparison to the somatic cells, we show here that the proTAME-induced arrest in these cells does not require SAC activity. Our results revealed important differences between mammalian oocytes and early embryos and somatic cells in their requirements of SAC for APC/C inhibition. In comparison to the somatic cells, oocytes and embryos show much higher frequency of aneuploidy. Our results are therefore important for understanding chromosome segregation control mechanisms, which might contribute to the premature termination of development or severe developmental and mental disorders of newborns.

## 1. Introduction

In both mitosis and meiosis, a transition from metaphase to anaphase requires the activation of a large multisubunit E3 ubiquitin ligase called anaphase promoting complex/cyclosome (APC/C) [1]. The activation of APC/C in somatic cells, as well as in oocytes, is controlled by the activity of the spindle assembly checkpoint (SAC), which facilitates the formation of the mitotic checkpoint complex (MCC) composed of BUB1 related kinase 1 (BUBR1), mitotic arrest deficient 2 (MAD2), budding uninhibited by benzimidazole 3 (BUB3) and cell division cycle 20 (CDC20) proteins on unattached kinetochores [2,3,4]. Only when all kinetochores are attached to the spindle and a tension between sister kinetochores is established does the production of MCC complex cease, which leads to APC/C activation by the released CDC20.

Recently, in a screen for small molecules capable of blocking cell cycle progression, a new inhibitor with the ability to prevent CYCLIN B degradation was identified [5]. Results from the same laboratory later showed that the tosyl-l-arginine methyl ester (TAME, or in a cell-permeable prodrug form called proTAME) blocks APC/C activation by its CDC20 and cadherin 1 (CDH1) activators [6]. The mechanism of TAME inhibition of APC/C is not completely understood, but it seems that TAME in part blocks the ability of free CDC20 to bind to the APC/C and also promotes the autoubiquitination of already prebound CDC20 [7].

It has also been shown that the metaphase arrest caused by proTAME requires SAC activity and that the RNAi knockdown of MAD2 shortened proTAME-induced mitotic arrest in HeLa cells, although the brief exposure of mitotic cells to the drug does not cause kinetochore attachment defects [6]. Consistently with the above, the dependency of proTAME blocking on SAC activity was also confirmed in another study, which revealed that prolonged proTAME arrest in mitosis causes cohesion fatigue and eventually the inappropriate attachment of sister chromatids to the spindle and the subsequent reactivation of SAC [8].

The inhibitory effect of proTAME on APC/C activity also seems to have great therapeutic potential. In combination with other inhibitors, proTAME was shown to be efficient in overcoming resistance caused by the hyperphosphorylation of CDH1 in glioblastoma cells [9], polo-like kinase 1 (PLK1)-based drug resistance in ovarian cancer cells [10] and CDC20-based resistance in diffuse large B-cell lymphoma [11].

In this study, we tested the effect of proTAME on APC/C activation in meiosis I in mouse and bovine oocytes and also in the mitosis of early mouse cleavage embryos. Using live cell imaging, we also studied whether the SAC activity is required for proTAME arrest in mouse oocytes. Our results showed that proTAME induces arrest in mouse and bovine oocytes, albeit only at higher doses in the latter. We also show here that the metaphase arrest induced by this drug does not require SAC activity. However, our results revealed that this drug dose-dependently affects morphological parameters of the spindle in oocytes and in embryos. In contrast to the somatic cells, the arrest in oocytes and embryos is not reversible.

## 2. Results

### 2.1. ProTAME Prevents Anaphase Entry in Mouse and Bovine Oocytes and also in Mouse 2-Cell Embryos

**Figure 1:**
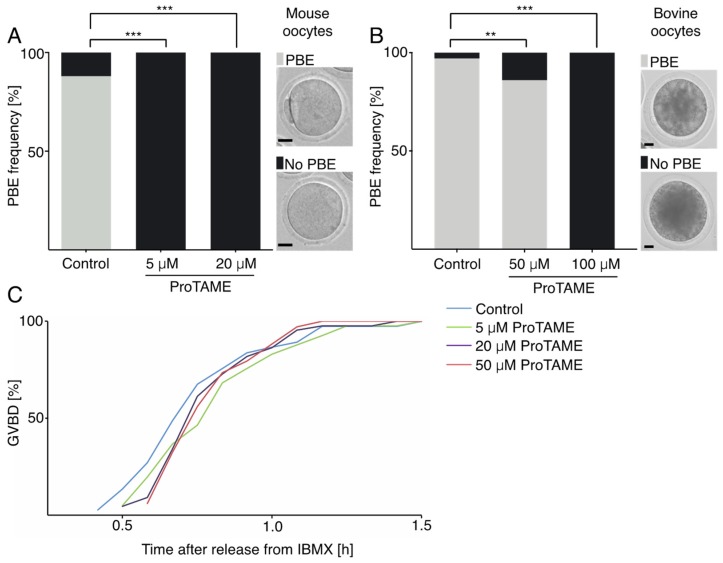
The impact of prodrug tosyl-l-arginine methyl ester (proTAME) on the meiotic maturation of mouse and bovine oocytes. (**A**) The frequency of polar body extrusion (PBE) in mouse untreated oocytes (*n* = 43) and oocytes treated with 5 μM (*n* = 42) and 20 μM (*n* = 44) proTAME was scored. Oocyte maturation was monitored by live cell microscopy and 88% of cells in the control group and 0% in 5 μM and 20 μM proTAME underwent PBE. Data were obtained in two independent experiments. The right side panel shows representative examples of oocytes with and without PB. Scale bar: 20 μm. The difference between the control group and both 5 μM and 20 μM proTAME is statistically significant (α < 0.05; *** *p* < 0.0001). (**B**) The frequency of PBE in bovine untreated oocytes (*n* = 97) and oocytes treated with 50 μM (*n* = 92) and 100 μM (*n* = 83) proTAME was scored. PBE was scored after 20 h of maturation. A total of 97% of control cells, 86% of cells in 50 μM and 0% of cells in 100 μM proTAME underwent PBE. Data were obtained in two independent experiments. The right side panel shows representative examples of oocytes with and without PB. Scale bar: 20 μm. The difference between the control and 50 μM proTAME is statistically significant (α < 0.05; ** *p* = 0.0080); the difference between the control and 100 μM proTAME is also statistically significant (α < 0.05; *** *p* < 0.0001). (**C**) Scoring of germinal vesicle breakdown (GVBD) in mouse untreated oocytes (light blue *n* = 37) and oocytes treated with 5 μM (green, *n* = 41), 20 μM (dark blue *n* = 44) and 50 μM (brown, *n* = 34) proTAME. GVBD was monitored by live cell microscopy and data were obtained in two independent experiments.

**Figure 2:**
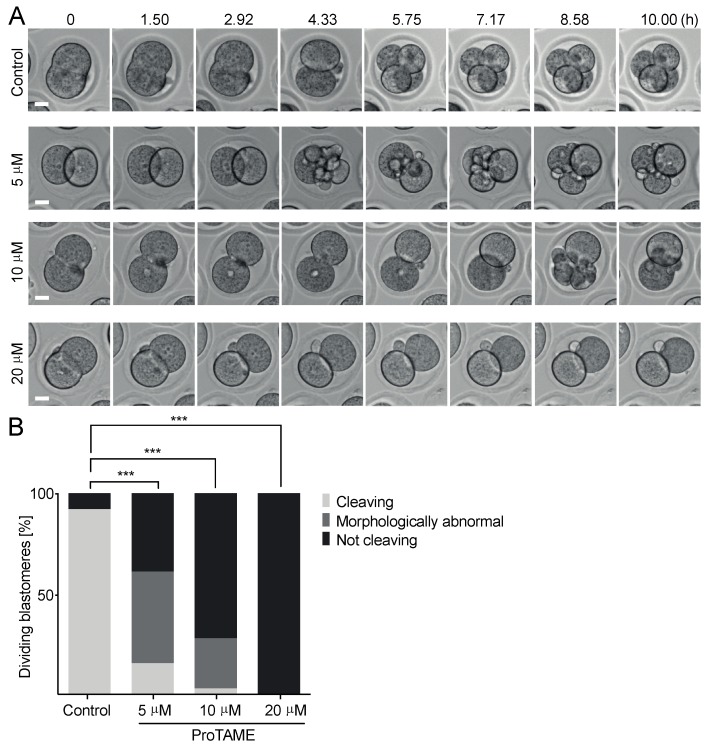
The impact of proTAME on the mitotic division of mouse two-cell embryos. (**A**) Frames from a time lapse microscopy experiment showing the cleavage of the untreated mouse embryo and embryos treated with 5 μM, 10 μM and 20 μM proTAME. Scale bar: 20 μm. (**B**) The frequency of cleaving, morphologically abnormal and not cleaving blastomeres was scored in control embryos (*n* = 60), embryos treated with 5 μM (*n* = 58), 10 μM (*n* = 60) and 20 μM (*n* = 60) proTAME. In the control group, 92% of blastomeres were cleaving with no morphological abnormalities, and 8% of blastomeres were not dividing. In 5 μM proTAME, 15% of blastomeres were cleaving with no morphological abnormalities, 45% of blastomeres showed morphological abnormalities and 40% of blastomeres were arrested. In 10 μM proTAME, 3% of blastomeres were cleaving, 25% of blastomeres showed morphological abnormalities and 72% of blastomeres were arrested. In 20 μM proTAME, 100% of blastomeres were arrested. Data were collected in two independent experiments. The difference between the control group and 5 μM, 10 μM, and 20 μM proTAME is statistically significant (α < 0.05; *** *p* < 0.0001).

It was shown previously that anaphase in somatic cells and also in mouse embryos is delayed or completely prevented by the addition of small molecule proTAME into the culture media [6,12,13]. In our experiments, we first tested various concentrations of proTAME and their impact on metaphase to anaphase transition in mouse meiosis I oocytes. Oocytes were isolated into M16 culture media containing 3-isobutyl-1-methylxanthine (IBMX), which blocked germinal vesicle break down (GVBD). Then, the cells were released into M16 media without IBMX containing 0, 5 and 20 μM concentrations of proTAME and transferred to the widefield microscope equipped with an incubator. The polar body extrusion (PBE) was monitored for the subsequent 16 h. Our results revealed that both concentrations—5 and 20 μM proTAME—prevented anaphase I in virtually all cells (Figure 1A). We also tested whether proTAME is capable of blocking metaphase–anaphase transition in bovine oocytes. For this experiment, bovine oocytes were cultured in media with various concentrations of proTAME. Our preliminary experiments indicated that, in contrast to the mouse oocytes, 5 and 20 μM proTAME concentrations were insufficient to arrest cells in metaphase I. With increased concentration of proTAME to 50 μM, 14% of cells remained arrested in metaphase I, and in 100 μM proTAME, 100% of oocytes were synchronized (Figure 1B), which means that bovine oocytes require a 20× higher concentration of proTAME for efficient synchronization in meiosis I in comparison to the mouse oocytes. It was shown that proTAME is capable of inhibiting both APC/C activators, CDC20 and CDH1 [6]. Morpholino targeting or knockout mouse lines showed that CDH1 is involved in the control of resumption of meiosis and GVBD [14,15]. To test this, we exposed mouse oocytes to different levels of proTAME ranging from 5 to 50 μM in concentration (Figure 1C). Scoring of GVBD rates by time lapse microscopy showed that even the 50 μM concentration is unable to accelerate the GVBD, indicating that in oocytes, CDH1 might be more resistant to proTAME inhibition than CDC20. We further tested the ability of proTAME to inhibit APC/C activity in mouse two-cell embryos. In a recent report from the Greg FitzHarris laboratory, the authors showed that 10 μM proTAME was capable of preventing the development of mouse embryos beyond the two-cell stage [13]. We used live cell microscopy to monitor two-cell embryos cultured in media supplemented with 0, 5, 10 and 20 μM proTAME (Figure 2A,B). Our results showed that 10 and 20 μM proTAME arrested 72% and 100% of embryonic blastomeres in mitosis. A concentration of 5 μM was significantly less efficient, and only 40% of blastomeres remained arrested. We also noticed that embryos exposed to 5 and 10 μM proTAME frequently exhibited severe morphological defects, and abnormal divisions were observed in 74% and 88% of blastomeres undergoing division. In contrast to this, embryos in 20 μM proTAME remained arrested in the two-cell stage without any attempt to divide (Figure 2A,B).

### 2.2. ProTAME Arrest of Meiosis I in Mouse Oocytes is Due to the Inhibition of APC/C

**Figure 3:**
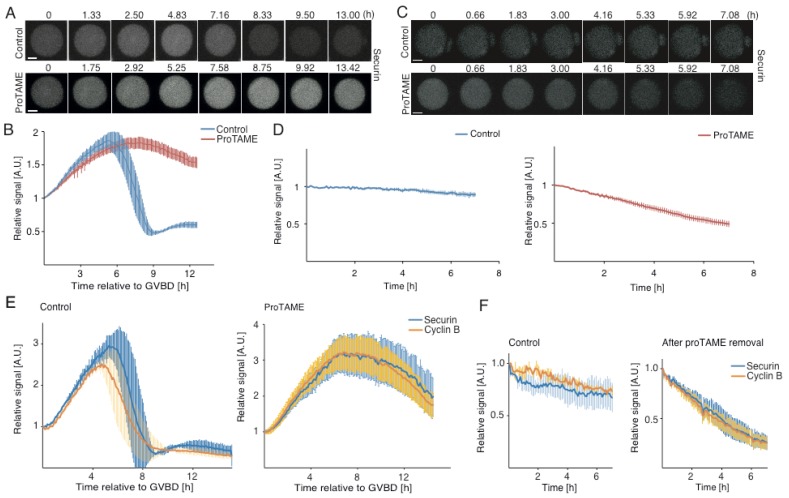
Oocytes arrested by proTAME show a low level of anaphase promoting complex/cyclosome (APC/C) activity. (**A**) Time frames from live cell microscopy showing the expression levels of microinjected SECURIN (grey) in various stages of meiotic maturation. Upper panels show the control cell, lower panels show the oocyte exposed to 5 μM proTAME. Scale bar: 20 μm. (**B**) Average SECURIN curves of control cells (blue, *n* = 5) and proTAME-treated cells (red, *n* = 11) with error bars. Time is relative to GVBD (a movie frame with GVBD or the first frame after GVBD represents time 0). Presented are representative results from a single experiment, which was repeated three times. (**C**) Time frames from live cell microscopy showing expression levels of microinjected SECURIN (grey) after the removal of proTAME from the culture media. Upper panels show the control cell, lower panels show cells previously exposed to 5 μM proTAME. Scale bar: 20 μm. (**D**) The left panel shows the average SECURIN curve with error bars of control cells (blue, *n* = 5) after proTAME removal; the right panel shows the average SECURIN curve with error bars of proTAME-treated cells (red, *n* = 11) after the removal of proTAME. The time is relative to the first frame after the removal of the inhibitor. Presented are representative results from a single experiment, which was repeated three times. (**E**) The left panel shows the average SECURIN (blue, *n* = 3) and CYCLIN B (yellow, *n* = 3) curves of control cells with error bars; the right panel shows the average SECURIN (blue, *n* = 6) and CYCLIN B (yellow, *n* = 8) curves of proTAME-treated cells with error bars. Time is relative to GVBD. Presented are results from a single experiment repeated twice. (**F**) The left panel shows the average SECURIN (blue, *n* = 3) and CYCLIN B (yellow, *n* = 2) curves of control cells, from the time point corresponding to proTAME removal, with error bars; the right panel shows the average SECURIN (blue, *n* = 6) and CYCLIN B (yellow, *n* = 7) curves of proTAME-treated cells after the removal of proTAME with error bars. Time is relative to the first frame after the removal of inhibitor. Presented are results from a single experiment; the experiment was repeated twice.

The activation of APC/C in mouse oocytes leads to the destruction of various substrates, including SECURIN [4]. In order to analyze the impact of exposure of oocytes to proTAME on APC/C activity, we microinjected tagged SECURIN cRNA into germinal vesicle (GV) oocytes; cells were then cultured in M16 media with and without proTAME, and expression levels of SECURIN were subsequently monitored for 14 h by live cell microscopy. Our results showed that in oocytes exposed to proTAME, the activation of APC/C was postponed and SECURIN levels were stabilized, in contrast to the control cells, in which SECURIN was destroyed as cells approached anaphase (Figure 3A,B). In order to test whether proTAME withdrawal would lead to the activation of APC/C and SECURIN destruction, proTAME was removed from the media and the SECURIN signal was monitored for an additional 7 h (Figure 3C,D). We observed that although the SECURIN levels decreased after the removal of proTAME, the dynamic of this process was incomparable to the SECURIN destruction in control cells approaching the anaphase (Figure 3B blue line), indicating that the APC/C was not fully activated. To verify our results, we used another APC/C substrate CYCLIN B1 (Figure 3E,F). Cells were microinjected with either SECURIN or CYCLIN B cRNAs and then monitored by live cell microscopy. Our results revealed that in control cells, both SECURIN and CYCLIN B were destroyed with similar dynamics during metaphase to anaphase transition (Figure 3E, left panel). Furthermore, both molecules were stabilized in the presence of proTAME (Figure 3E, left panel). After the removal of proTAME from the culture media, the levels of SECURIN and CYCLIN B decreased with similar dynamics (Figure 3F), which was again slower than in cells approaching anaphase with fully active APC/C (Figure 3E).

### 2.3. ProTAME-Induced Arrest of Oocytes in Meiosis I Is Irreversible

**Figure 4:**
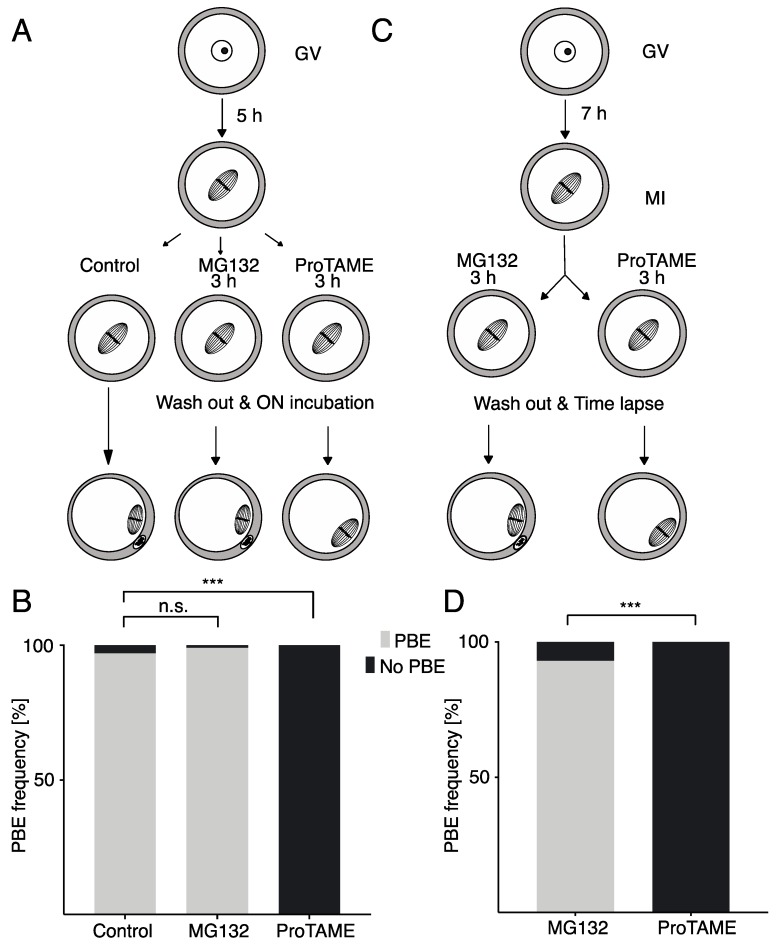
ProTAME-induced arrest in mouse oocytes is irreversible. (**A**) Germinal vesicle (GV) oocytes were matured for 5 h and then divided into three groups: the control group, MG132-treated group and proTAME-treated group. The control group was incubated overnight in M16 media. MG132 and proTAME groups were exposed to inhibitors for 3 h, washed out and incubated overnight in M16 media. The frequency of PBE was scored following overnight (ON) incubation. (**B**) The frequency of PBE in control oocytes (*n* = 70), oocytes in 10 μM MG132 (*n* = 79) and oocytes in 5 μM proTAME (*n* = 81) was scored. A total of 97% of control cells, 99% of cells after MG132 and 0% of cells after proTAME extruded polar bodies (PBs). Data were obtained in three independent experiments. The difference between the control and MG132 is not statistically significant (α < 0.05; *p* = 0.6008). The difference between the control and proTAME is statistically significant (α < 0.05; *** *p* < 0.0001). (**C**) GV oocytes were matured for 7 h and then divided into two groups; one was treated with MG132 and the other with proTAME for 3 h and subsequently cultured in M16 without inhibitors while PBE was monitored by live cell microscopy ON. (**D**) The frequency of PBE in oocytes after 10 μM MG132 (*n* = 43) and 5 μM proTAME (*n* = 51) was scored. A total of 93% of cells after MG132 and 0% of cells after proTAME extruded PBs. Data were obtained in two independent experiments. The difference between both groups is statistically significant (α < 0.05; *** *p* < 0.0001).

ProTAME is an efficient inhibitor of cell cycle progression [6]; however, to our knowledge there is no detailed information about the reversibility of this arrest. We therefore compared the reversibility of synchronization by proTAME and proteasome inhibitor MG132 using mouse oocytes. In this series of experiments (Figure 4A), GV oocytes were matured for 5 h in M16 media without inhibitors. Then, cells were divided into three groups and cultured in M16 media with no inhibitor, with 10 μM MG132 and with 5 μM proTAME for 3 h. After this treatment, cells were released into inhibitor-free M16 media and cultured overnight with PBE scoring the next morning. Our results showed that MG132 treatment was fully reversible, whereas 100% of cells exposed for 3 h to proTAME remained arrested in meiosis I (Figure 4B). These experiments were repeated with a prolonged interval of oocyte maturation prior to the addition of inhibitors (Figure 4C). Instead of 5 h, cells were cultured for 7 h, which should provide them with enough time to activate the APC/C (this takes place normally around 6 h after the disassembly of the nuclear membrane called GVBD (Figure 3B). However, the results (Figure 4D) were similar, confirming the irreversibility of proTAME arrest in mouse oocytes.

**Figure 5:**
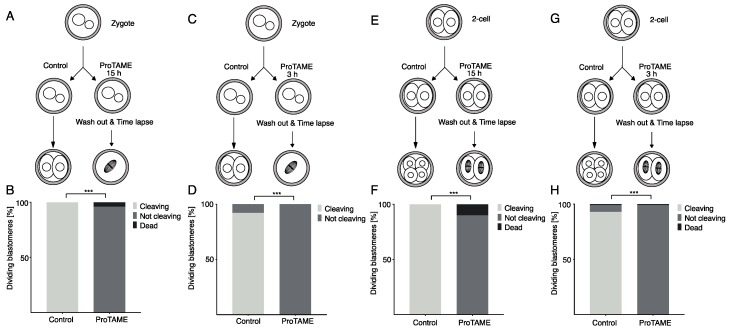
ProTAME-induced arrest in mouse embryos is irreversible. (**A**) Zygotes isolated 18–21 h post hCG stimulation were divided into two groups: the control group and proTAME-treated group. ProTAME-treated zygotes were exposed to the inhibitor for 15 h and washed out, and their development was monitored by live cell microscopy. (**B**) The frequency of developing zygotes in the control group (*n* = 68) and in the group after 20 μM proTAME treatment (*n* = 80). A total of 100% of control zygotes and 0% of zygotes after 20 μM proTAME treatment developed to the two-cell stage, and 4% zygotes after 20 μM proTAME treatment were dead. Data were obtained in two independent experiments. The difference between the control group and proTAME is statistically significant (α < 0.05; *** *p* < 0.001). (**C**) Zygotes isolated 21 h post hCG stimulation and 26 h post hCG were divided into two groups: the control group and proTAME-treated group. ProTAME-treated zygotes were exposed to the inhibitor for 3 h and washed out, and their development was monitored by live cell microscopy. (**D**) The frequency of developing zygotes in the control group (*n* = 25) and in the group after 20 μM proTAME treatment (*n* = 25). A total of 92% of control zygotes and 0% of zygotes after 20 μM proTAME treatment developed to the two-cell stage. The difference between the control group and proTAME is statistically significant (α < 0.05; *** *p* < 0.001). Presented are results from a single experiment. (**E**) Two-cell embryos isolated 41 h post hCG stimulation and 48 h post hCG cells were divided into two groups: the control group and proTAME-treated group. ProTAME-treated embryos were exposed to the inhibitor for 15 h and washed out, and development was monitored by live cell microscopy. (**F**) The frequency of dividing two-cell blastomeres in the control group (*n* = 173) and in the group after 20 μM proTAME treatment (*n* = 158). A total of 100% of control blastomeres were cleaving and 0% of blastomeres after 20 μM proTAME treatment were cleaving; 10% of blastomeres after 20 μM proTAME treatment were dead. The difference between the control group and proTAME is statistically significant (α < 0.05; *** *p* < 0.001). Data were obtained in two independent experiments. (**G**) Two-cell embryos isolated 41 h post hCG stimulation and 50 h post hCG cells were divided into two groups: the control group and proTAME-treated group. ProTAME-treated embryos were exposed to the inhibitor for 3 h and washed out, and development was monitored by live cell microscopy. (**H**) The frequency of dividing two-cell blastomeres in the control group (*n* = 100) and in the group after 20 μM proTAME treatment (*n* = 100). A total of 93% of control blastomeres and 0% of blastomeres after 20 μM proTAME treatment were cleaving, while 1% of blastomeres in each group were dead. The difference between the control group and proTAME is statistically significant (α < 0.05; *** *p* < 0.001). Data were obtained in two independent experiments.

We also tested the reversibility of proTAME synchronization in zygotes and two-cell embryos using time lapse microscopy (Figure 5A–H). In both developmental stages, we used prolonged, 15 h exposure (Figure 5A,E), since our preliminary results showed that the unsynchronized population of two-cell embryos enter mitosis within an 11.5 h interval. The prolonged synchronization was compared to only 3 h exposure (Figure 5C,G). Both groups of cells were synchronized by 20 μM proTAME. Our results demonstrated that exposure to proTAME for 15 h (Figure 5B,F) or 3 h (Figure 5D,H) led to cell cycle arrest (Figure 5B,D,F,H). Surprisingly, embryos exposed to proTAME only for 3 h, which were mostly in interphase during this interval (Supplemental Figure S1A), also arrested in the following mitosis. Similarly to the oocytes, after the removal of the inhibitor from the culture media, embryos were unable to resume the cell cycle (Figure 5F,H).

### 2.4. ProTAME Affects Spindle Morphology in Oocytes and Embryos

**Figure 6:**
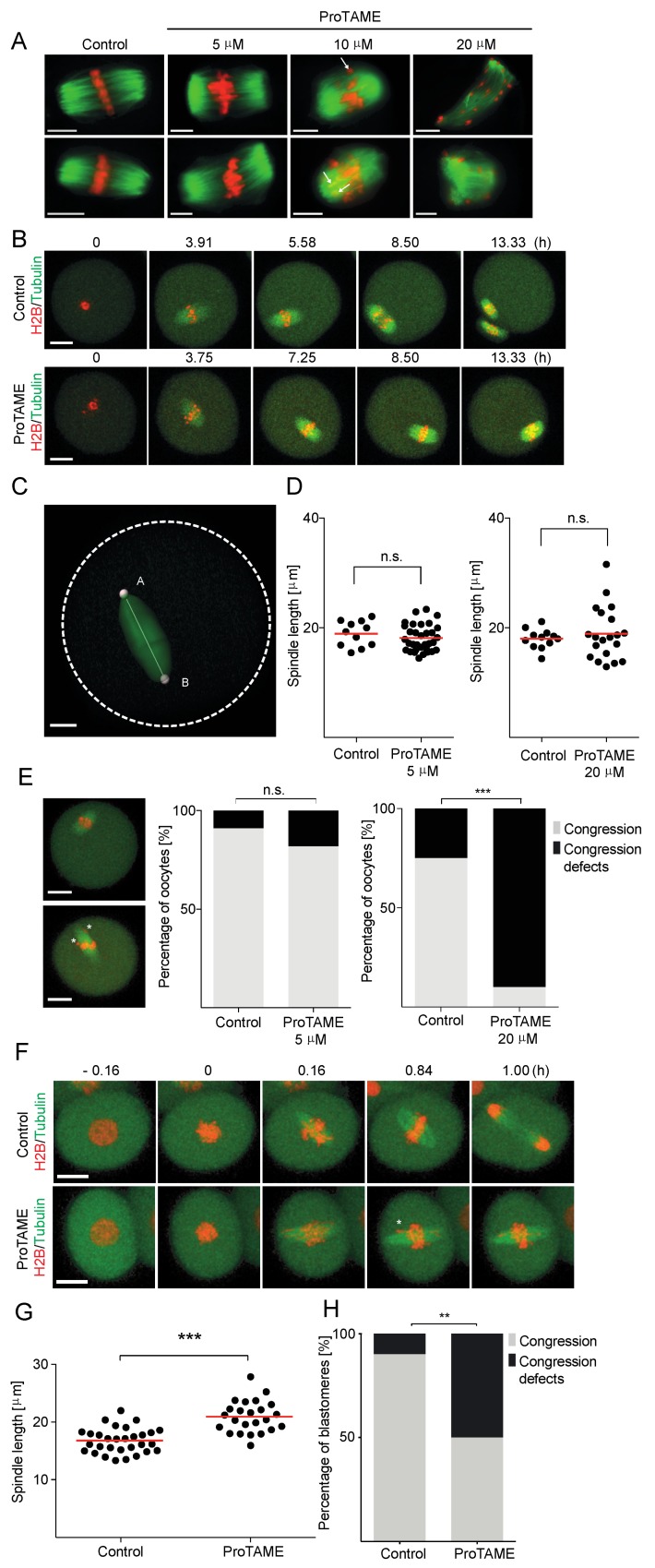
Effect of proTAME exposure on spindle morphology in mouse oocytes and mouse two-cell embryos. (**A**) Examples of spindle morphology of control oocytes and oocytes treated with 5, 10 and 20 μM proTAME. TUBULIN (green) was detected with an antibody recognizing acetylated TUBULIN, and DNA (red) was visualized with DAPI. Chromosomes separated from the metaphase plane are indicated by an arrowhead. Scale bar: 10 μm. (**B**) Time frames from a live cell imaging experiment of oocytes microinjected with TUBULIN (green) and HISTONE H2B (red) ORFs fused to fluorescent proteins. Upper frames show the control oocyte, the lower panel shows the oocyte cultured in 5 μM proTAME. Scale bar: 20 μm. (**C**) The example of spindle length measurement. A movie frame with control oocyte microinjected with TUBULIN (green). Scale bar: 10 μm. (**D**) The left scatter plot shows the spindle length in control (*n* = 11) and 5 μM proTAME-treated oocytes (*n* = 33). Data were obtained in three independent experiments. The difference between the control and 5 μM proTAME-treated cells is not significant (*p* value = 0.3368). The right scatter plot shows the spindle length in control (*n* = 12) and 20 μM proTAME-treated oocytes (*n* = 20). The red line represents the mean. Data were obtained in two independent experiments. The difference between the control and 20 μM proTAME-treated cells is not significant (*p* value = 0.5291). (**E**) Movie frames on the left show examples of an oocyte with normal congression (upper panel) or an oocyte with chromosome congression defects (lower panel), scale bar: 20 μm. Congression defects are indicated by asterisks. The left chart shows the frequency of chromosome congression defects in control (*n* = 11) and 5μM proTAME-treated oocytes (*n* = 33). A total of 9.09% of control oocytes and 18.18% of oocytes treated with 5 μM proTAME exhibited congression defects. Data were obtained in three independent experiments; the difference between the control group and 5 μM proTAME was not statistically significant (α < 0.05; *p* = 0.6594). The right chart shows the frequency of chromosome congression defects in control (*n* = 12) and 20 μM proTAME-treated oocytes (*n* = 20) with 25.00% of control and 90.00% of proTAME treated oocytes showing congression defects. Data were obtained in two independent experiments; the difference between the control group and 20 μM proTAME was statistically significant (α < 0.05; *** *p* < 0.001) (**F**) Time frames from live cell imaging experiment with two-cell stage embryos microinjected with TUBULIN (green) and HISTONE H2B (red) fused to fluorescent proteins. Upper frames show the control embryo, the lower panel shows the embryo cultured in 20 μM proTAME, scale bar: 20 μm. Congression defects are indicated by asterisk. (**G**) Scatter plot showing spindle length in control blastomeres (*n* = 30) and 20 μM proTAME-treated blastomeres (*n* = 24). The difference between the control group and proTAME was statistically significant (α < 0.05; *** *p* < 0.001). The red line represents the mean. Data were obtained in two independent experiments. (**H**) Frequency of chromosome congression defects in control (*n* = 30) and 20 μM proTAME-treated blastomeres (*n* = 24). A total of 10% of control blastomeres and 50% of blastomeres treated with 20 μM proTAME showed congression defects. The difference between the control group and proTAME was statistically significant (α < 0.05; ** *p* < 0.010).

It was previously shown that proTAME at levels inducing metaphase arrest does not affect spindle morphology or chromosome attachment [6]. In contrast to the centrosomal origin of the spindle in mitosis, in meiosis, the spindle is assembled from the microtubule organizing centers (MTOCs), which then cluster on the spindle poles, and MTOC-dependent spindle assembly continues in mice, even in early embryos [16,17]. It also seems that meiotic spindles tend to form multipolar structures, at least in humans [18,19]. In order to address whether the exposure to proTAME affects spindle morphology, we first tested several concentrations of this drug and the effect on the mouse female meiosis I spindle (Figure 6A). For this, we matured mouse oocytes in M16 media with 0, 5, 10 and 20 μM concentrations of proTAME overnight. Our data showed that a 5 μM concentration has no visible effect on spindle morphology, whereas 10 and 20 μM proTAME affects the spindle after overnight exposure. To verify that the basic spindle morphology at a 5 μM concentration is largely unaffected, GV oocytes were microinjected with cRNAs encoding HISTONE H2B, TUBULIN and SECURIN fused to various fluorescent proteins. Oocytes were divided into two groups and matured with or without 5 μM proTAME in the media while monitoring the spindle morphology. There was no visible damage to the spindle, although oocytes exposed to 5 μM proTAME did not extrude polar bodies within the duration of the experiment in contrast to the cells in the control group (Figure 6B). Based on the average SECURIN expression in the control oocytes, we obtained the timepoint before the activation of APC/C, which is manifested by the initiation of SECURIN destruction, and measured the spindle length at this timepoint using Imaris software (Figure 6C). Our results showed that there was no significant difference in the length of the spindle between the control group and the 5 μM proTAME group (Figure 6D, left panel). Although our assays were not sensitive enough to reveal possible subtle changes in spindle morphology—for example, perturbations of microtubule–kinetochore attachments—the results suggest that proTAME at 5 μM concentration has no major effect on spindle morphology in oocytes. We also performed similar experiments comparing control cells and cells exposed to 20 μM proTAME (Figure 6D, right panel). Again, the difference between the spindle length in the control oocytes and oocytes exposed to 20 μM proTAME was not statistically significant; however, we noticed that the variability of the spindle length in the proTAME group is much higher than in the control group. Additionally, the spindle structure in proTAME group also dramatically changed its shape with increasing time (Supplementary movies S1–S3). In control oocytes and oocytes exposed to 5 μM and 20 μM proTAME, we also scored the congression defects represented by chromosome visibly separated from the equatorial plane in the last frame before anaphase (Figure 6E, left and right charts). Our results showed that the 5 μM proTAME had no significant effect on chromosome congression, whereas increasing the proTAME concentration to 20 μM dramatically affected the frequency of chromosome congression defects (Figure 6E, right chart). To assess the situation in mouse early embryos, two-cell stage embryos were microinjected with cRNAs encoding HISTONE H2B and TUBULIN fused to fluorescence protein tags (Figure 6F). The length of the spindle and congression defects were scored in the last frame before anaphase in control embryos or at a similar timepoint in embryos exposed to 20 μM proTAME (Figure 6G,H). In comparison to oocytes exposed to 20 μM proTAME, the spindle length in the embryos mainly extended instead of changing its length in both directions. Furthermore, although the congression defects in two-cell embryos exposed to 20 μM proTAME increased in comparison to the control embryos (Figure 6H), they were not so frequent as in oocytes in 20 μM proTAME. Our results showed that although the proTAME concentration sufficient to induce metaphase I arrest in oocytes has little effect on the spindle, increasing proTAME concentration to levels required for metaphase arrest in embryos significantly increased the frequency of spindle and chromosome congression defects.

### 2.5. ProTAME-Induced Arrest of Mouse Oocytes and Embryos Does Not Require SAC

**Figure 7:**
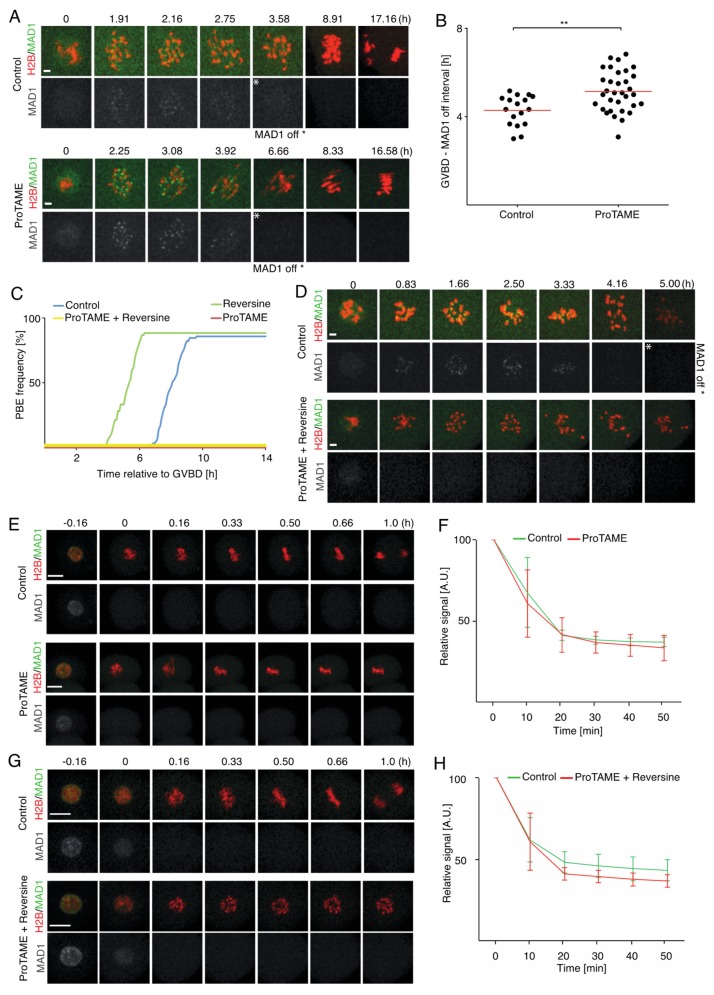
ProTAME-induced arrest in mouse oocytes and two-cell embryos does not require SAC activity. (**A**) Time frames from live cell imaging experiment showing MAD1 localization: upper panel—control cell; lower panel—cell exposed to 5 μM proTAME. MAD1 (green) and HISTONE H2B (red) visualized by fusion into fluorescent proteins. The time frame with MAD1 removal (MAD1 off) from chromosomes is indicated with an asterisk. 0 h = GVBD. Scale bar: 5 μm. (**B**) Scatter plot showing GVBD–MAD1 off interval in control oocytes (*n* = 17) and 5 μM proTAME-treated oocytes (*n* = 33). Data were obtained in three independent experiments. The red line represents the mean. The difference between control and proTAME-treated cells is statistically significant (α < 0.05; ** *p* < 0.010). (**C**) Cumulative graph showing the timing of polar body extrusing (PBE) during meiosis I in control oocytes (blue, *n* = 84), in oocytes treated with 500nM reversine (green, *n* = 60), in oocytes treated with 5 μM proTAME (red, *n* = 20) and in oocytes treated with 5 μM proTAME + 500 nM reversine (yellow, *n* = 58). Data were collected in two independent experiments. (**D**) Time frames from live cell imaging experiment using GV oocytes showing MAD1 localization, where upper panels represent the control cell, and lower panels represent the cell exposed to 5 μM proTAME + 500 nM reversine. MAD1 (green) and histone H2B (red) visualized by fusion to fluorescent proteins. The time frame with MAD1 removal (MAD1 off) from chromosomes is indicated with an asterisk (control). 0 h = GVBD. Scale bar: 5 μm. (**E**) Time frames from live cell imaging experiment with two-cell embryos showing MAD1 localization; upper panels represent the control cell, lower panels represent the cell exposed to 20 μM proTAME. MAD1 (green) and HISTONE H2B (red) visualized by fusion to fluorescent proteins. 0 h = NEBD. Scale bar: 20 μm. Data were collected in a single experiment with seven control and eight proTAME-treated two-cell embryos. (**F**) Average MAD1 density measured in the vicinity of chromosomes in blastomeres of control embryos (green, *n* = 11) and in blastomeres of proTAME-treated embryos (red, *n* = 13) with error bars. 0 min = the last frame before NEBD. (**G**) Time frames from the live cell imaging experiment with two-cell embryos showing MAD1 localization; upper panels represent the control cell, lower panels represent the cell exposed to 20 μM proTAME and 500nM reversine. MAD1 (green) and HISTONE H2B (red) visualized by fusion to fluorescent proteins. 0 h = NEBD. Scale bar: 20 μm. Data were collected in a single experiment with six control and eight proTAME + Reversine treated two-cell embryos (**H**) Average MAD1 density measured in the vicinity of chromosomes in blastomeres of control embryos (green, *n* = 11) and in blastomeres of proTAME + Reversine-treated embryos (red, *n* = 10) with error bars. 0 min = the last frame before NEBD.

It was shown previously that, in somatic cells, SAC activity is required for proTAME-induced metaphase arrest and that the abolishing of SAC activity by MAD2 knockdown released cells from this block [6]. Later, it was shown that the prolonged proTAME arrest in mitosis causes a loss of sister chromatid cohesion, which in turn activates SAC [8]. Mitotic arrest deficient 1 (MAD1) is regularly used as a marker of SAC activity in mouse oocytes and embryos, and its presence on kinetochores indicates active SAC [13,20]. We therefore used MAD1 overexpression to monitor the SAC activity in mouse oocytes arrested in proTAME. GV oocytes were microinjected with cRNAs encoding HISTONE H2B and MAD1 fused to fluorescent proteins. Cells were then matured in M16 media with or without 5 μM proTAME, and the expression and localization of MAD1 was monitored during meiosis I by live cell imaging (Figure 7A). Our results showed that in cells cultured with and without proTAME, MAD1 was initially present on chromosomes, and after some time it disappeared from chromosomes in both groups (Figure 7A). Scoring the time between GVBD and MAD1 departure from the chromosomes showed that this interval is longer in proTAME-treated oocytes; however, all oocytes in our analysis eventually lost the MAD1 signal (Figure 7B). This clearly demonstrates that SAC is not required to maintain the proTAME-induced meiosis I arrest in mouse oocytes. To assess whether SAC is required initially for establishing proTAME arrest, we treated cells simultaneously with proTAME and the monopolar spindle 1 (MPS1) inhibitor reversine. MPS1 is required in oocytes for SAC function, namely for MAD2 localization on kinetochores and the correct congression and segregation of chromosomes [21], and the exposure of oocytes to reversine during meiosis I causes the acceleration of meiosis I [22]. Our results confirmed that reversine alone accelerated polar body extrusion, but in combination with proTAME, PBE was blocked, similar to the case with proTAME only (Figure 7C). Using MAD1 overexpression assay revealed, that in contrast to the control cells, oocytes treated simultaneously with reversine and proTAME lost the MAD1 signal from chromosomes coincidentally with GVBD or shortly after GVBD (Figure 7D and Supplementary movies S4 and S5). This indicates that the proTAME arrest in oocytes does not require SAC signaling on the kinetochores, even at the beginning. Since, in somatic cells, the SAC seems to be required for proTAME arrest [6], we used two-cell embryos, representing a transition between meiosis and mitosis, and analyzed whether SAC is required for proTAME arrest in embryonic blastomeres of two-cell embryos. In a preliminary experiment, embryos were treated with reversine, and our results showed that the duration of mitosis was on average 38 min in reversine-treated cells in comparison to 50 min in control cells (Supplemental Figure S1B). We also noticed that almost all embryos exposed to reversine showed lagging chromosomes and massive congression defects. Assessing the effect of proTAME on MAD1 localization in two-cell embryos showed that the MAD1 signal disappeared from the vicinity of the chromosomes shortly after nuclear envelope breakdown (NEBD), and the association with chromosomes was thereafter only transient in both control embryos as well as in embryos treated with proTAME (Figure 7E,F). A similar situation was observed in embryos treated with a combination of reversine and proTAME; cells lost their MAD1 signal from the chromosomes shortly after NEBD, similarly to the control embryos (Figure 7G,H). This suggests that abolishing SAC activity on kinetochores in two-cell embryos has no effect on establishing or maintaining proTAME arrest, similar to the case of oocytes.

## 3. Discussion

We show here that proTAME is capable of inducing meiosis I arrest in two mammalian species, although bovine oocytes require a 20x higher concentration of the drug than the mouse oocytes. This difference might be partially due to the variability in the composition of the culture media used for mouse and bovine oocytes. In fact, when we cultured mouse oocytes in bovine maturation media, we observed only 24% of arrested cells in 5 μM proTAME, in comparison to 100% in M16 media with the same level of proTAME (Figure 1). However, in 50 μM proTAME in bovine media, 100% of mouse oocytes were arrested (in the control group of mouse oocytes cultured in bovine media without proTAME, 96.8% of cells matured), whereas only 14% of bovine oocytes were arrested in the same conditions (Figure 1). The lower permeability of bovine oocytes for proTAME or lower sensitivity of APC/C to proTAME inhibition in bovine oocytes might also contribute to this difference. Currently, we are investigating both possibilities.

We confirmed that mouse embryos are sensitive to proTAME and that the majority of them respond with metaphase arrest when exposed to 10 μM or higher concentrations, similar to the recently published results [13]. In our experiments with two-cell embryos, lower proTAME concentrations (5 and 10 μM) frequently induced various morphological defects during division in comparison to the control embryos. This might increase the possibility of embryo damage or the perturbation of subsequent development.

Our results further showed that in mouse oocytes arrested in proTAME, the APC/C substrates SECURIN and CYCLIN B were stabilized in comparison to cells undergoing anaphase, which is consistent with the low level of APC/C activity [4]. After the removal of the inhibitor from the culture media, the activity of APC/C was still very low in comparison to the metaphase–anaphase transition during meiosis I, and the characteristics of the SECURIN and CYCLIN B degradation profiles typical for this transition were missing. This indicates that after the removal of proTAME, cells were unable to fully activate APC/C and enter anaphase. The observed decrease of SECURIN and CYCLIN B signals was probably caused by factors other than by the APC/C activity itself; for example, by the cessation of translation. This is in accordance with our results, which revealed that the proTAME arrest in oocytes and embryos is irreversible. Our experiments also revealed that to establish permanent arrest, the timing of the drug exposure relative to the SAC or APC/C activity is not crucial and cells are arrested in mitosis also after exposure during interphase.

Importantly, our results demonstrated that SAC activity is not required to maintain proTAME arrest in mouse oocytes. Based on MAD1 localization in live oocytes, we showed that the SAC is turned off with only a short delay in comparison to control cells, despite the presence of proTAME in the culture media. Similarly, in two-cell embryos, the proTAME arrest was established despite the departure of MAD1 from the chromosomes. This is different than in somatic cells, in which SAC seems to be required for proTAME-induced arrest, and it was shown that MAD2 RNAi depletion abolished this block [6]. We also show here that SAC activity is not required to induce proTAME arrest in oocytes and two-cell embryonic blastomeres, even initially. This was achieved using reversine, a specific inhibitor of MPS1, which in oocytes prevents the loading of MAD2 on kinetochores and causes premature anaphase entry [21,22]. We show here that oocytes exposed to the combination of reversine and proTAME failed to recruit MAD1 to the kinetochores, which is consistent with abolished SAC, and also failed to extrude their polar bodies, demonstrating that the APC/C was inhibited. The role of SAC in prolonged proTAME-induced mitotic delay in somatic cells was explained by cohesion fatigue and the reduction of cohesin on the chromosomes, leading to sister chromatid cohesion errors and SAC reactivation [8]. It was demonstrated that the prophase pathway, which in somatic cells removes the majority of the cohesin from chromosome arms, plays also important role in proTAME-induced arrest [8]. However, this pathway is absent in mammalian meiosis [23], and the removal of cohesin from chromosome arms in mouse female meiosis I is solely dependent on SEPARASE; even prolonged arrest in the absence of SEPARASE does not lead to a reduction of cohesion [23,24]. This could explain our results using MAD1, which showed that SAC is not reactivated in the presence of proTAME. Furthermore, although proTAME exposure caused spindle elongation and frequent chromosome congression defects in two-cell embryos, we were unable to detect a reaccumulation of MAD1 on kinetochores. This might exclude the participation of SAC on proTAME arrest even after prolonged exposure, which in somatic cells caused the cohesion fatigue reactivation of SAC [8]. Although it is still unclear whether APC/C-induced proteolysis is required for SAC inactivation in somatic cells, we show here that this activity is clearly not required for SAC attenuation in oocytes. This might be important for understanding the differences between the regulation of APC activity in oocytes and in somatic cells. The inability of oocytes to recover from even a short exposure to proTAME and the relative stability of SECURIN and CYCLIN B after proTAME removal both suggest that the APC/C in oocytes is irreversibly affected by this drug. Additionally, it is conceivable that such exposure might affect the stability or localization of important APC/C regulators such as CDC20.

## 4. Materials and Methods

### 4.1. Animals, Isolation of Oocytes and Embryos and Cell Cultivation

CD-1 mice were purchased from the Animal Breeding and Experimental Facility, Faculty of Medicine, Masaryk University, Brno, Czech Republic; BDF1 males were purchased from Anlab, Prague, Czech Republic. CD-1/BDF1 mice were obtained by crossing CD-1 female mice and BDF1 male mice. For all experiments, at least three-month-old females were used. All animal work was conducted according to Act No 246/1992 Coll. on the protection of animals against cruelty and was approved by the Central Commission for Animal Welfare, approval ID 51/2015, 24 June 2016. To collect embryos, mice were stimulated with 5 IU of pregnant mare serum gonadotropin (PMSG; Merck, Kenilworth, NJ, USA) followed 44–48 h later by stimulation with 5 IU of human chorionic gonadotropin (hCG; Merck) and mated with BDF1 males. Zygotes and two-cell embryos were isolated 18–21 and 41–45 h after hCG stimulation by the manual rupturing of the oviduct in M2 medium (Merck) and subsequently cultured until the next manipulation or inhibitor treatment in KSOM + AA medium (Caisson Labs, Smithfield, UT, USA) covered with mineral oil (Merck) at 37 °C, 5% CO_2_. Zygotes were incubated for 5 min with 1% protease (Merck) for cumulus cell removal. GV-stage oocytes were isolated by the technique described previously [25]. Bovine oocytes were isolated from ovaries, which were transferred to the laboratory from a local slaughterhouse in phosphate-buffered saline (PBS) within 7 h of slaughter. Cumulus–oocyte complexes (COCs) were isolated from 2–8 mm ovarian follicles by aspiration and washed twice in washing medium containing 90% of MPM (modified Parker’s medium, home-made) and 10% of fetal bovine serum (FBS, ThermoFisher Scientific, Waltham, MA, USA). The cumulus cells were removed by vortexing. The oocytes without cumulus cells were than cultured at 39 °C, 5% CO_2_ in a maturation medium containing 90% of MPM (home-made), 10% of FBS (ThermoFisher Scientific), 1% of follicle stimulating hormone (FSH, Stimufol, Reprobiol, Ouffet, Belgium) and 50 ng/mL of epidermal growth factor (EGF, Merck) until the next manipulation or inhibitor treatment. After 20 h of incubation for PBE scoring, the oocytes were fixed in 3.7% paraformaldehyde (Merck) for 20 min at room temperature (RT) and permeabilized with 0,1% Triton (Merck) for 15 min at RT. Oocytes were mounted on microscope slides in Vectashield mounting medium with DAPI (Vector Laboratories, Burlingame, CA, USA).

### 4.2. Microinjection of GV Oocytes and Embryos

The microinjection of mouse GV oocytes was performed by a technique previously described [26]. The microinjection of embryos was performed in M2 medium (Merck). In microinjection experiments, the following cRNAs fused with fluorescent proteins were used: SECURIN-CFP, CYCLIN B1-Venus, TUBULIN-Venus, MAD1-Venus, HISTONE H2B-mCherry and HISTONE H2B-mPlum. cRNAs for microinjection were diluted in RNase free water to a concentration of 2–4 ng per μL.

### 4.3. Treatment with Inhibitors

In experiments, isolated/microinjected cells were treated with 5, 10, 20, 50 or 100 μM proTAME inhibitor (Bio-Techne R&D Systems, Minneapolis, MN, USA) diluted in KSOM + AA medium (Caisson Labs)/M16 medium (Merck)/bovine maturation medium (home-made) according to the experiment. Control cells were cultivated in KSOM + AA medium (Caisson Labs)/M16 medium (Merck)/bovine maturation medium (home-made) without inhibitors. In the GVBD timing test, the oocytes were transferred from M16 medium (Merck) supplemented with 100 μM 3-isobutyl-1-methylxanthine (IBMX, Merck) to M16 medium (Merck) with different concentrations of ProTAME (Bio-Techne R&D Systems) or without an inhibitor and GVBD was monitored. In the inhibitor reversibility test, the mouse oocytes were treated with 5 μM proTAME (Bio-Techne R&D Systems) or 10 μM MG132 (Merck) for 3 h, and mouse embryos were treated for ~15 h or ~3 h with 20 μM proTAME (Bio-Techne R&D Systems); then, the oocytes and embryos were washed out and PBE (oocytes)/development (embryos)/mitosis entry (embryos) was monitored on a widefield microscope, or oocytes were incubated overnight in an incubator and PBE was scored on a stereomicroscope after 18 h. To treat cells with both proTAME (Bio-Techne R&D Systems) and reversine (Merck) inhibitors, the following concentrations were used: 5 μM concentration of proTAME (Bio-Techne R&D Systems) for oocytes, 20 μM concentration of ProTAME (Bio-Techne R&D Systems) for embryos, and 500 nM concentration of reversine (Merck) for both oocytes and embryos.

### 4.4. Immunodetection

The immunofluorescence protocol was modified from [24]. Zona pellucida was removed by briefly incubating the oocytes in acid Tyrode solution (Merck). Cells were fixed in 3.7% paraformaldehyde (Merck) for 60 min, permeabilized with 0.1% Triton X-100 (Merck) for 15 min and blocked for 15 min at RT. The antibodies mouse anti-acetylated α-TUBULIN (1:500 dilution, Merck) and Alexa Fluor 488 goat anti-mouse (1:500 dilution, ThermoFisher Scientific) were used. Vectashield with DAPI (Vector Laboratories) was used for mounting.

### 4.5. Microscopy and Live Cell Imaging

The live imaging assay of microinjected oocytes was performed on a Leica SP5 confocal microscope equipped with an EMBL incubator (37 °C, 5% CO_2_) (EMBL, Heidelberg, Germany) and with an HCX PL APO 40×/1.1 water immersion objective. The 458 nm, 514 nm and 561 nm excitation wavelengths and hybrid detectors were used for the detection of CFP, Venus, mCherry and mPlum fluorescent proteins. The Live Image Assay for concentration and reversibility experiments was performed on a Leica AF 6000 inverted fluorescence microscope, equipped with an EMBL incubator (37 °C, 5% CO_2_) and HC PL FLUOTAR 10×/0.30 without immersion, and the transmitted light was used for detection. For bovine PBE scoring, the cells were scanned on a Leica SP5 confocal microscope equipped with an HCX PL APO CS 40×/1.30 oil immersion. An excitation wavelength of 405 nm and hybrid detector were used for the detection of the DAPI signal, and transmitted light was used for the detection of PBs. The oocytes after immunofluorescence staining were scanned on a Leica AF 6000 inverted fluorescence microscope, equipped with an HCX PL APO 100.0×/1.40 oil immersion objective. Filter cubes A (excitation filter BP 360/40) and green fluorescent protein (GFP, excitation filter 470/40) were used for the detection of DAPI and Alexa Fluor 488. All microscopy equipment was from Leica company (Leica, Wetzlar, Germany).

### 4.6. Image Analysis and Statistical Analysis

ImageJ 1.52m (ImageJ, National Institutes of Health, Bethesda, MA, USA), LAS AF 2.7.3 (http://www.leica-microsystems.com) and Imaris software 9.2.1 (www.bitplane.com) were used for image analysis. Graphpad Prism 5 for Mac OS X (https://www.graphpad.com/scientific-software/prism/) was used for the statistical testing of data. Specific statistic tests used in the article are the D’Agostino & Pearson omnibus normality test, one-tailed Student’s t-test, Fisher’s exact test and Chi-square test. For the quantification of the SECURIN and CYCLIN B signals, the expression levels after the subtraction of the background were normalized to the level at GVBD, similar to the procedure used previously [4].

## 5. Conclusions

In conclusion, our data suggest that proTAME-induced arrest in mouse oocytes and early embryos does not require activity of the SAC. It is still not clear whether in somatic cells SAC is required continuously or only after prolonged incubation of cells in proTAME. However, we clearly excluded any role of SAC in proTAME induced APC/C based arrest in mouse oocytes and embryos and therefore our results suggest that the control of APC/C activity by SAC in these cells might be different than in somatic cells.

## Supplementary Materials

Supplementary materials can be found at https://www.mdpi.com/1422-0067/20/18/4537/s1. Supplement Figure S1: (A) Percentage of zygotes (*n* = 25) and 2-cell blastomeres (*n* = 90) which were in interphase during proTAME treatment. 96% of zygotes and 69% of 2-cell blastomeres were in interphase during proTAME incubation (NEBD scored in first frame of time lapse after removal of proTAME). Results of one experiment with zygotes and two experiments with 2-cell blastomeres. (B) Mitosis length in control (*n* = 9) and reversine treated (*n* = 12) 2-cell blastomeres. The difference between the control group and reversine is statistically significant (α < 0.05; ** *p* < 0.010). Supplemental movies S1, S2 and S3: All movies show oocytes injected with H2B-mCherry, TUBULIN-Venus and SECURIN-CFP cRNAs, movie S1 shows representative control oocyte, movies S2 and S3 show representative oocytes treated with 20 μM proTAME. Time is relative to GVBD. Supplemental movie S4 and S5: Both movies show the first 145 min after GVBD in oocytes injected with MAD1-Venus and H2B-mCherry cRNAs, movie 4 shows control oocyte, movie 5 shows oocyte treated with 5 μM proTAME.

## Abbreviations

APC/C	Anaphase promoting complex/cyclosome
BUBR1	BUB1 related kinase 1
BUB3	Budding uninhibited by benzimidazole 3
CDC20	Cell division cycle 20
CDH1	Cadherin 1
COCs	Cumulus–oocyte complexes
GV	Germinal vesicle
GVBD	Germinal vesicle breakdown
IBMX	3-isobutyl-1-methylxanthine
MAD1	Mitotic arrest deficient 1
MAD2	Mitotic arrest deficient 2
MCC	Mitotic checkpoint complex
MI	Metaphase I
MPS1	Monopolar spindle 1
MTOCs	Microtubule organizing centers
NEBD	Nuclear envelope breakdown
ON	Overnight
PBE	Polar body extrusion
PLK1	Polo-like kinase 1
ProTAME	Prodrug tosyl-l-arginine methyl ester
RT	Room temperature
SAC	Spindle assembly checkpoint
