# Supplementary material for: ProTAME Arrest in Mammalian Oocytes and Embryos Does Not Require Spindle Assembly Checkpoint Activity
Source: Int J Mol Sci. 2019 Sep 13;20(18):4537. doi: 10.3390/ijms20184537 (PMC6770151; doi:10.3390/ijms20184537)

Supplement 1

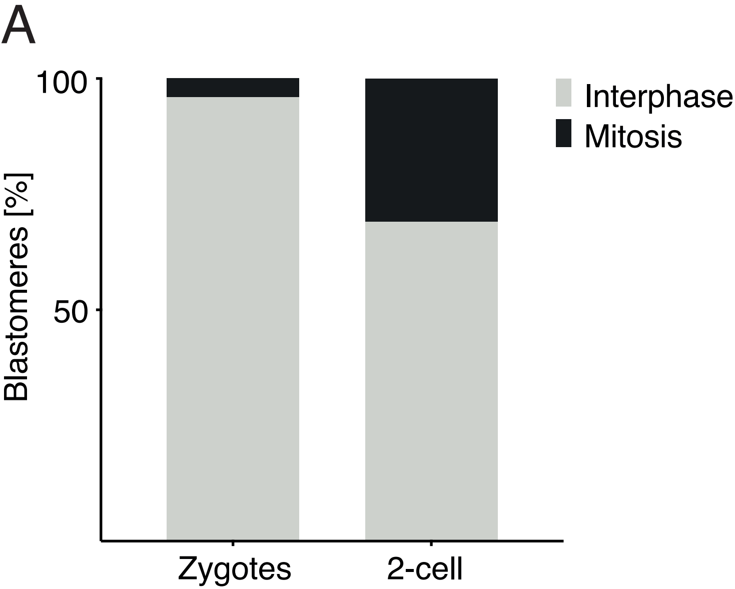

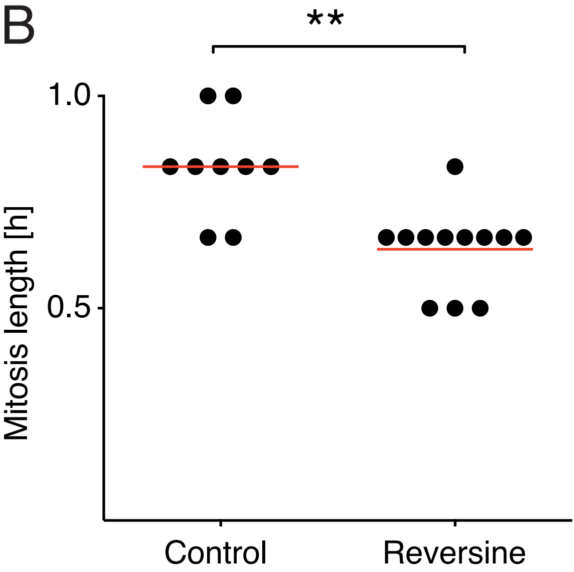

Supplement: Supplementary file 1 [file Supplement_1.pdf › Supplement_1.pdf]
